# The protective effect of hydroalcoholic extract of *Ephedra pachyclada* leaves on ovarian damage induced by cyclophosphamide in rat: An experimental study

**DOI:** 10.18502/ijrm.v21i8.14018

**Published:** 2023-09-20

**Authors:** Hassanali Abedi, Mahnaz Nemati, Bahare Ebrahimi, Maryam Dehghani, Elmira Mikaeiliagah, Pegah Abdollahzadeh, Aref Ghanaatpishe, Nazanin Shafiee Jahromi, Hossein Kargar Jahromi

**Affiliations:** ^1^Research Center for Noncommunicable Diseases, Jahrom University of Medical Sciences, Jahrom, Iran.; ^2^Amir Oncology Hospital, Shiraz University of Medical Sciences, Shiraz, Iran.; ^3^Shiraz Geriatric Research Center, Shiraz University of Medical Science, Shiraz, Iran.; ^4^Department of Biology, Ardabil Branch, Islamic Azad University, Ardabil, Iran.; ^5^Stem Cell and Tissue Engineering Laboratory, Department of Orthopaedics, West Virginia University, Morgantown, USA.; ^6^Student Research Committee, Jahrom University of Medical Sciences, Jahrom, Iran.; ^7^Department of Microbiology, Jahrom Branch, Islamic Azad University, Jahrom, Iran.

**Keywords:** Ephedra, Cyclophosphamide, Ovary, Follicles, Hydroalcoholic extract.

## Abstract

**Background:**

Cyclophosphamide (CP) is an anticancer drug that acts as an alkylation agent after metabolism in the liver. CP has toxic effects on the body's cells, especially the reproductive system's function, and causes infertility. Moreover, medicinal plants have few side effects and are psychologically acceptable to patients.

**Objective:**

This study aimed to investigate the impact of *Ephedra pachyclada* hydroalcoholic extract (EPHE) on ovarian tissue and hypothalamic-pituitary-gonadal axis in rats treated with CP.

**Materials and Methods:**

In this experimental study, 48 adult female Wistar rats (180-200 gr, 9-10 wk) were randomly assigned to 6 experimental groups (n = 8/each): (a) control; (b) sham; (c) CP; (d) CP+250 mg/kg EPHE; (e) CP+500 mg/kg EPHE; (f) CP+1000 mg/kg EPHE. On the 29 ${}^{\text{th}}$ day of the experiment, serum was collected; serum concentration of the luteinizing hormone, follicle-stimulating hormone, estrogen, progesterone, and antioxidant activity were measured. The number of ovarian follicles were also counted.

**Results:**

In the CP groups, serum concentrations of follicle-stimulating hormone and luteinizing hormone significantly increased, and estrogen and progesterone significantly decreased (p ≤ 0.05). EPHE significantly compensated for the complications caused by CP and 1000 mg/kg had the greatest effect. Antioxidant reduction by CP was significantly enhanced by EPHE, especially at higher doses (p ≤ 0.05). The number of primordial, primary, secondary, and Graafian follicles showed a significant decrease in CP groups and EPHE groups showed a significant increase compared to the CP. EPHE showed that the concentration of 1000 mg/kg was more effective than other doses (p ≤ 0.05).

**Conclusion:**

In addition to proving the effect of EPHE on the hypothalamic-pituitary-gonadal axis, our investigation showed antioxidant properties, which can be an effective factor in CP-treated rats.

## 1. Introduction

Cyclophosphamide (CP) is an anticancer drug, which acts as an alkylation agent and inhibits DNA replication by alkylating. This drug is widely used in clinics to treat autoimmune disorders and ovarian cancer. The chemotherapy process by CP in the reproductive system is highly toxic, especially in the sexual organs. One of its most important complications is the changes in the function of the reproductive system and ultimately infertility, which can affect the fertility rate in men and women. It also has detrimental effects on folliculogenesis, leading to irreversible ovarian damage or inflammation (1). CP metabolism occurs in the liver. CP undergoes the effects of microsomal enzymes in the liver, then decomposes into its active metabolites such as Acrolein and phosphoramide mustard. Overdose of CP leads to inhibiting immune responses and stopping tumor growth by weakening the body's defense mechanism (2).

CP probably inhibits cancerous cell proliferation. Studies have also shown that it can cause apoptosis in cancer cells in rat fetuses, lung cells, and, rat thymus (3). *Ephedra pachyclada* is a plant of the Ephedraceae family. The plants of this family are famous because of many ephedrine alkaloids, the most important of them are ephedrine and pseudoephedrine (4), they have many healing properties, and have been used for thousands of years in treating respiratory diseases such as asthma, bronchitis, and allergies (4, 5). *Ephedra* also has anti-inflammatory, antioxidant, and blood sugar-lowering effects, as well as imitating the sympathetic nervous system (6, 7). Among the alkaloids, ephedrine is an active ingredient in this plant, and pseudoephedrine is also found in some species of *Ephedra*. Ephedrine and pseudoephedrine make up more than 80% of the alkaloids in the *Ephedra* plant. In addition to ephedrine, other secondary metabolites, such as flavonoids, tannins, etc., are also present in the *Ephedra* plant as alkaloids (8). A study on one of the species of *Ephedra* found that hydroalcoholic extract had a better effect than aqueous extract, and it has stronger antioxidant, anti-inflammatory, and antiproliferative capacities than aqueous extract (9).

Oxidative stress and inflammation have been shown to have a close relationship and free radicals can cause inflammation (10). Although the effectiveness of different species of *Ephedra* on ovarian tissue and the destructive effects of CP have been confirmed in several studies, there are few reports of the plant's effect on ovarian-related hormones and its tissue changes. Therefore, the effect of *Ephedra pachyclada* hydroalcoholic extract (EPHE) on ovarian tissue in rats treated with CP and its modifications has been investigated in this study.

## 2. Materials and Methods

### Collection and extraction of *Ephedra*

This plant was collected from the areas around Shiraz and then the extraction was done by the Kelvinger device. The stems of the *Ephedra pachyclada* plant were dried (30 C) and powdered. Crude plant powder was soaked with an ethanol/water mixture (70/30) for 7 days at 35 ± 5 C. The mixture was then filtered using a sterile cloth sheet and dried under reduced pressure at < 45 C with a rotary evaporator to obtain a dark red residue with a yield of 7%. Before use, the extract was stored in universal bottles and refrigerated at 4 C (11).

### Experiment design

48 healthy female Wistar rats (9-10 wk, 180-200 gr) were used in this experimental study, and the rats were randomly (random numbers table) divided into 6 groups (n = 8/each). The rats were kept in the animal laboratory of Jahrom University of Medical Science, Jahrom, Iran for a week to adapt (4 rats per cage). 12 hr light/dark cycle, with the humidity of about 50-55% were considered. According to previous studies, EPHE was prescribed with concentrations of 250, 500, and 1000 mg per kg body weight. The EPHE was fed to animals by gavage. The prescribed concentration of CP was determined as 5 mg/kg body weight (11). Therefore, the Sham and experimental groups in this study include the following groups. The control group (no treatment), Sham group (distilled water, by gavage) and experimental groups; experimental group 1 (5 mg/kg CP monohydrate, Sigma-Aldrich, St, Louis, MO), and experimental 2, 3, and 4 received 5 mg/kg CP and EPHE with concentrations of 250, 500, and 1000 mg per kg body weight respectively, like the rest of groups for 28 consecutive days at 8-10 AM.

### Biochemical analysis

At the end of the study (day 29), after weighing the animals, blood was drawn directly from the animal's heart with a 5 cc syringe (under anesthesia using Ketamine and Xylazine). Then, their serum was collected by the centrifugal device (for 15 min and 3000 rpm) and kept at -20 C until the experiment's time. To measure the gonadotropin-releasing hormone, follicle-stimulating hormone (FSH), luteinizing hormone (LH), estrogen, and progesterone hormones, ELIZA kits (Shanghai crystal day biotech Co., China) for rats made were used.

### Histological examination

For histological examination, left and right ovarian tissue were removed, placed informalin 10%, and after 48 hr, paraffin sections (5, and 20 um thickness) were prepared. 12 sections of each ovary were selected and stained by the Haemotoxylin and Eosin (H&E) method. Finally, primordial, primary, secondary, Graafian, and atretic follicles were examined with light microscopy. To count the follicles, the optical dissector method, mikrokator, and special counting frame were used. The frames were placed on the microscopic fields (100 × objective), up to a depth of 5 µm each slice. The desired depths were obtained with a mikrokator (MT12, Germany) (12).

### Analysis of oxidative stress markers

#### Lipid peroxidation and glutathione peroxidase (GSH-Px) assay 

Plasma Mmalondialdehyde (nM/mL) and GSH-Px activity were measured using commercial kits.

#### Total antioxidant capacity (TAC) and total oxidant status (TOS) measurements

TAC and TOS were measured with commercially available kits (Rel Assay Diagnostics, Turkey). Measurements were performed according to the manufacturer's instructions.

### Ethical considerations

In this research, all ethical issues regarding how to work with laboratory animals have been considered by Jahrom University of Medical Science Ethics committee, Jahrom, Iran (Code: IR.JUMS.REC.1396.019).

### Statistical analysis

At first, the normality of the data was measured using the Shapiro-Wilk normality test. The data were analyzed by a one-way ANOVA test and Duncan Post Hoc using SPSS version 21 software (SPSS, Chicago, IL, USA). P ≤ 0.05 were considered statistically significant. All data were reported as mean ± SD.

## 3. Results

### Biochemical examinations

**Figure 1:**
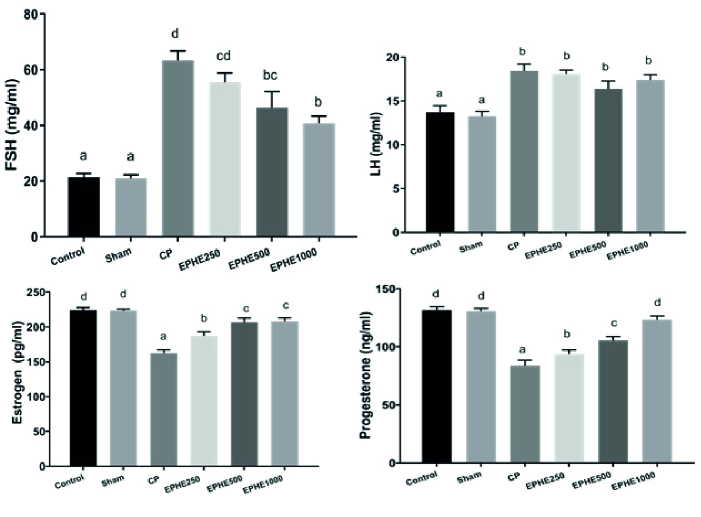
Comparison of mean FSH, LH, estrogen, and progesterone concentrations in different groups based on the Duncan test. Each group means that at least one shared letter has no significant difference. P < 0.05 is considered statistically significant. EPHE: *Ephedra pachyclada* hydroalcoholic extract.

No significant difference in FSH, LH, estrogen, and progesterone concentrations were observed between groups. Serum concentrations of FSH and LH were significantly increased in all experimental groups compared to the control, and serum concentrations of estrogen and progesterone significantly decreased. Also, mean serum concentrations of FSH and LH in experimental groups CP + 250 mg/kg EPHE, CP + 500 mg/kg EPHE, and CP + 1000 mg/kg EPHE showed a significant decrease compared to the CP group, and estrogen and progesterone showed a significant increase. A comparison of groups that received both CP and EPHE showed that the concentration of 1000 mg/kg had more effect than other concentrations on reducing FSH and increasing progesterone and estrogen, and the concentration of 500 mg/kg had more effect on reducing LH and increasing estrogen than other concentrations but the decrease in LH was not significant. Generally, the concentration of 1000 mg/kg of EPHE showed the greatest effect compared to other concentrations in most variables (Figure 1).

### Histopathological examinations

**Figure 2:**
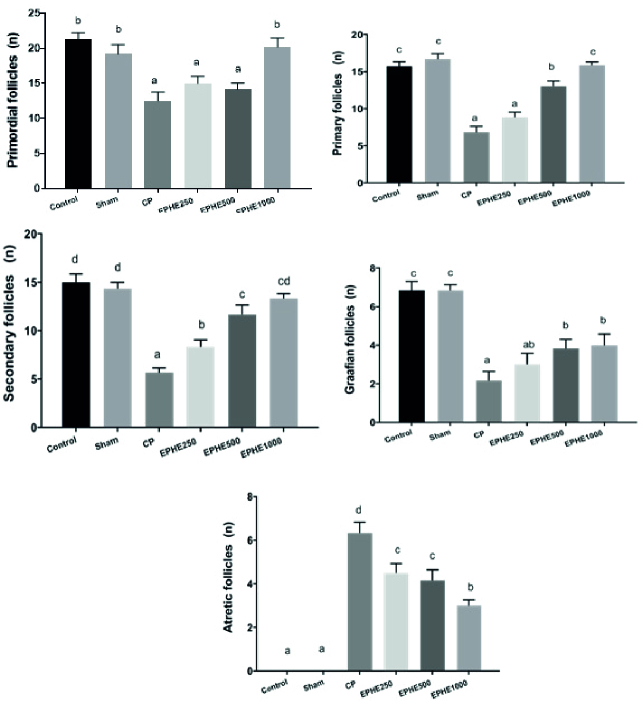
Comparison of the mean number of primordial follicles, primary follicles, secondary follicles, Graafian follicles, and Atretic follicles in different groups based on the Duncan test. Each group means that at least one shared letter has no significant difference. P < 0.05 is considered statistically significant. EPHE: *Ephedra pachyclada* hydroalcoholic extract.

No significant difference was observed between Sham and control groups regarding the mean number of primordial, primary, secondary, Graafian, and atretic follicles. In experimental groups, 1, 2, and 3, primordial, primary, and secondary follicles were significantly reduced compared to the control group. Graafian follicles were significantly reduced in all experimental groups, and a significant increase was observed in atretic follicles compared to the control group. Also, the mean number of primary, secondary, and Graafian follicles in experimental groups 3 and 4 showed a significant increase compared to the CP group (experimental 1). The mean number of atretic follicles in all experimental groups compared to the CP group showed a significant decrease at the level of p < 0.05. A comparison of groups receiving both CP and EPHE showed that the concentration of 1000 mg/kg had more effect than other concentrations in increasing or decreasing the number of follicles (Figure 2).

### Evaluation of oxidant/antioxidant status

**Figure 3:**
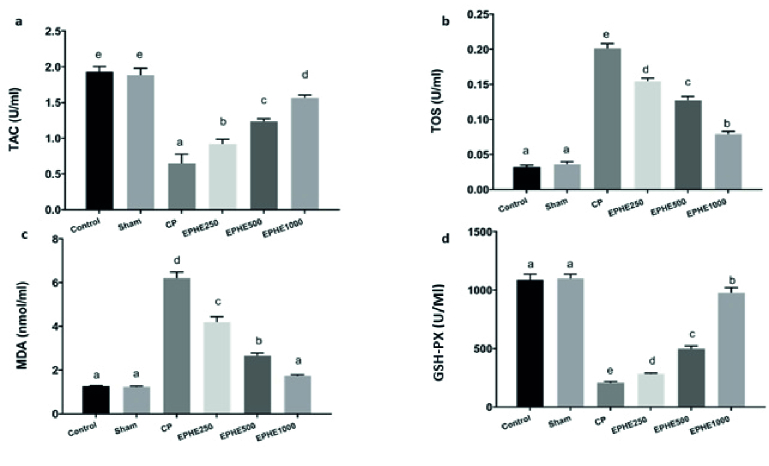
Comparison of the oxidant/antioxidant status in different groups based on the Duncan test. Each group means that at least one shared letter has no significant difference. P < 0.05 is considered statistically significant. EPHE: *Ephedra pachyclada* hydroalcoholic extract. MDA: Malondialdehyde.

As shown in figure 3, CP significantly decreased the TAC (p < 0.01) and increased TOS (p < 0.05) at the end of the study (day 29). EPHE (250, 500, and 1000 mg/kg) treatment elevated the (p < 0.01) and attenuated the TOS (p < 0.05) when compared with the CP group. After 29 days in the CP group, the Malondialdehyde levels showed a significant increased (p < 0.01), while the GSH-Px activity level was reduced (p < 0.05) in comparison with the control and sham groups. Using EPHE (250, 500, and 1000 mg/kg) could reduce the Malondialdehyde levels and increase GSH-P activity significantly in comparison with CP. Interestingly, EPHE (1000 mg/kg) group brought these factors to the control and Sham level.

## 4. Discussion

The process of chemotherapy with CP in the reproductive system is highly toxic and has adverse effects on folliculogenesis, which lead to irreversible damage to the ovaries (13). The results of one study showed that CP consumption in female rats significantly increased the mean serum concentrations of LH and FSH.

The number of atretic follicles significantly decreased the serum concentration of estrogen and the number of primordial, primary, secondary, and Graafian follicles. This destruction of the follicle is one of the main causes of ovarian failure and infertility caused by chemotherapy, confirming the destructive effects of CP on these parameters and is consistent with other studies (14). In other studies that checked out the effect of CP on male mice, the levels of LH, FSH, progesterone, testicular weight, and the count and motility of sperm decreased significantly compared to the control group (15). The gonadotropin hormone regulates folliculogenesis, so the decrease in the number of follicles and the increase in the number of atretic follicles can be caused by a decrease in gonadotropin hormones following the toxic effects of CP.

Many plants have antioxidant properties and are involved in antimutagenic cellular activities. Since the progression of cancer and chemotherapy has a close relationship with oxidative stress, compounds with antioxidant properties can be helpful to treat and prevent this (16). In this study, it is possible that different concentrations of EPHE due to its antioxidant properties, which are mostly due to the presence of ephedrine and pseudoephedrine, can modify the changes made by CP and play a role in the treatment of induced reproductive disorders in female rats. This property can also be caused by herbacetin glycosides, an active biological compound in the *Ephedra* plant (5). In addition to ephedrine and pseudoephedrine alkaloids, other studies had reported that *Ephedra* had other metabolits, such as flavones, carboxylic acid, and volatile terpenes.

Various studies have shown the antimetastatic and anticancer effects of this plant, its mechanism was inhibition and suppression of cancer cells motility, as well as the inhibition of the hepatocyte growth factor due to the movement of cancer cells (5). The results of the present study showed that its effect was related to the maximum concentration of *Ephedra*, that is 1000 mg/kg, which indicates that hydroalcoholic extractis dose-dependent, as it performs significantly better.

Acrolein, a metabolite derived from CP, is responsible for toxic effects such as cell death and apoptosis by disrupting the body's antioxidant pathways and producing free radicals (17). There are several theories about the mechanism of action of CP: CP affects cell division and induces intra and inter-standard cross-link, and also because of its cytotoxic and mutagenic properties, it is considered an alkylating agent. CP connects to two molecular strands of DNA, break it down, and finally inhibits protein synthesis. CP induced reactive molecules affect nucleophilic groups of DNA, especially 7-n-guanine (18). CP especially affects the ovary; it causes severe ovarian damage, altered folliculogenesis, steroid production, and a reduced number of follicles. CP causes ovarian dysfunction by increasing apoptosis, altering folliculogenesis, and affecting steroids (19). CP is converted to 4-hydroxy CP by cytochrome P-450 enzymes in the liver, which accelerates the pathway of apoptosis (20).

Acrolein interferes in the antioxidant system and produces oxygen-free radicals. This metabolite also inhibits the GSH - Px system. Following inhibition, the cell nucleus in the ovary is destroyed, and the cells become apoptotic. CP can increase apoptotic cells in the ovary and disrupt the function of the oxidative stress system in the ovaries. The most important oxidative system in the ovaries is the GSH - Px system, where its activity is reduced by CP, and increased cell damage due to increase in the ROS levels. Jeelani et al., reported that exposure to reactive oxygen species produced after exposure to CP reduces ovum quality (21). Some studies show that CP toxicity causes ovarian damage through direct or indirect effects. The direct effect leads to the death of the ovum, and the indirect effect targets the somatic cells, in which both induce apoptosis (22).

CP activating PI3K/PTEN/Akt pathway can affects oocytes, pregranulosa cells, and primordial follicles (23).

The expression of B-cell lymphoma 2 family genes, which play an important role in the apoptosis pathway, is disrupted in ovarian cells exposed to CP. In addition, CP activates the caspase 3 enzyme, which is one of the main and effective enzymes of the downstream apoptotic pathway (24, 25). In another study, in CP-treated mice, the anti-apoptotic factor B-cell lymphoma-extra-large decreased, and the proapoptotic factor BAX increased.

Another theory about CP is that it induces diminished ovarian reserve due to the rapid absorption of primordial follicles (26). Another cause of increased apoptosis following CP intake is an increase in the body's autoimmune activity, which causes the ovaries to invade and destroy the rest of the oocytes and, eventually the remaining follicles (27). Most follicles become atretic during the menstruation cycle and cause antigen production. These antigens do not normally trigger an immune attack. It is thought that, as a result of stimulation, regulatory T cells suppress and control autoreactive T cells induced by their antigens (28).

Therefore, it is assumed that CP stimulates the production of autoreactive T cells by causing extensive apoptosis, which results in the loss of the remaining oocytes. CP is also known as an immune system suppressor, and studies show that it also destroys the regulatory T cells (29). *Ephedra* can inhibit prostaglandin estradiol biosynthesis through pseudoephedrine, in this way it can induce analgesic and anti-inflammatory effects (30).

All studies on the effects of CP on ovarian tissue confirm the results of this study. Since CP is a widely used drug in chemotherapy and treatment of refractory diseases, it is also an alkylating agent, which has mutagenic and cytotoxic properties and can damage the body tissues through oxidative stress. Hence, taking antioxidants during chemotherapy or other procedures along with this drug effectively reduces oxidative stress and tissue detoxification. According to this study, it can be said that CP has harmful effects on ovarian tissue, secretion of sex hormones, and ovarian follicles in female rats.

In addition, EPHE improves the damages caused by CP, which can be effective in preventing the destructive effects of this drug, preserves the ovaries and follicles, and modifies sex hormones using its antioxidant effects on ovarian tissue. As more effects were observed in female rats, therefore it is said to be dose dependent. These doses may be different in humans. Therefore, this should be considered to generalize the results in humans.

## 5. Conclusion

In this study, CP as an alkylating agent caused destructive effects on ovarian tissue of rats, by disrupting the function of cells and sex hormones, and production of toxic metabolites and free radicals. However, EPHE, with antioxidant properties, reduced these side effects and improved it.

##  Conflicts of Interest

The authors declare that there is no conflict of interest.
